# Alterations of Cognition and Cerebral Ventricle Volume in Manic and Euthymic Pediatric Bipolar Disorder

**DOI:** 10.3389/fpsyt.2020.593629

**Published:** 2020-12-14

**Authors:** Liangfeng Kuang, Dong Cui, Qing Jiao, Yongxin Guo, Weifang Cao, Weijia Gao, Jianfeng Qiu, Linyan Su, Guangming Lu

**Affiliations:** ^1^Department of Radiology, Shandong First Medical University & Shandong Academy of Medical Sciences, Taian, China; ^2^Department of Child Psychology, The Children's Hospital, Zhejiang University School of Medicine, Hangzhou, China; ^3^Mental Health Institute of the Second Xiangya Hospital, Key Laboratory of Psychiatry and Mental Health of Hunan Province, Central South University, Changsha, China; ^4^Department of Medical Imaging, Jinling Hospital, Clinical School of Medical College, Nanjing University, Nanjing, China

**Keywords:** Pediatric bipolar disorder (PBD), mania, euthymia, ventricle volume, cognition

## Abstract

**Introduction:** It remains unknown whether volumetric alterations of ventricles are similar or not in pediatric bipolar disorder (PBD) among different mood states. The present study aims to estimate ventricular volumetric alteration of PBD patients in manic and euthymic status, as well as the relationship between this alteration and cognitive changes.

**Methods:** T1 magnetic resonance images were obtained from 20 manic PBD patients, 21 euthymic PBD patients, and 19 healthy controls (HCs). Ventricular volumes were automatically obtained via FreeSurfer 6.0 software. Ventricular volumes and cognitive indices were compared among the three groups, and the relationship between ventricular volumes and cognitive/clinical indices was analyzed.

**Results:** In contrast to HCs, manic and euthymic PBD patients exhibited decreased cognitive scores of the Stroop color-word test and the digit span subtest. Manic PBD subjects presented enlarged volumes in the bilateral ventricles, third ventricle, and whole ventricles, and euthymic PBD participants displayed increased volumes in the third ventricle, fourth ventricle, and whole ventricles. No significant differences in cognitive performance and ventricular volumes were found between PBD groups. No significant correlation was discovered between ventricular volumes and cognitive/clinical indices in both manic and euthymic PBD patients.

**Conclusions:** No significant differences in cognitive performance and ventricle volume were observed between euthymic and manic PBD groups, which may imply that the alterations are not specific to mood state. It may indicate structural and functional damage of corresponding brain circuits in euthymic PBD patients similar with that of manic PBD, which may provide clues to the diagnosis and treatment of euthymic PBD.

## Introduction

Pediatric bipolar disorder (PBD) is a severe psychiatric illness that recurrently attacks patients, marked by manic or hypomanic depressed episodes, and separated by euthymic periods. Manic PBD can feature extreme joy or rage, full vigorousness and inattention, difficultly falling asleep and reduced sleep needs, and racing thoughts and increased speech, while depressed PBD is characterized by inattention and suicidal tendencies, increased sleep needs, and emotional instability, and euthymic PBD generally shows no clinical psychiatric manifestations. With heritability of about 59% (1), bipolar disorder (BD) is reported to relate with considerably high prevalence and psychiatric comorbidity rate (2), high risk of suicide (3), and impaired cognition (4).

It has been found that BD patients exhibit alterations of ventricular volume (5). Some studies reported enlarged lateral ventricular volume in adult BD patients (6–10), while other researchers reported no difference in lateral ventricular volume between adult BD patients and healthy controls (HCs) (11, 12). Most studies have focused on ventricles' alterations in one mood state of BD. In the manic state, the third and lateral ventricle volumes in the first episode of adult BD (13) and the cerebral ventricular size in young male BD patients were shown to be increased (14). In euthymic adult BD, the width of the third ventricle exhibited an increase (15). Nevertheless, different results have also been reported, such as finding no lateral ventricle volume alteration in euthymic and manic BD adults (16–18). One study compared the width of the third ventricle in depressed, manic, and euthymic adult BD type I (BD I), and the researchers found increased width of the third ventricle in BD patients. No width difference of the third ventricle was displayed among the three patient groups (19). Several studies focused on ventricles in PBD and found increased lateral ventricle volume (20), enlarged lateral ventricles, and ventricle asymmetry in manic PBD subjects (21). The ratio of ventricle cerebrospinal fluid to cerebral total tissue volume, showing no difference between PBD and HCs, has also been reported (22). The above studies mostly involved one mood state of BD and have only reported alteration of the lateral and third ventricles. However, the difference in ventricular volume among different mood states and the relationship between ventricular volume alteration and cognitive tests remains unknown in PBD.

In the current study, we aimed to investigate alterations of the ventricular volumes of PBD patients in manic and euthymic states. According to prior research, we hypothesized that the volumes of four ventricles would be larger in PBD patients than those of the HCs. The current study was designed to: (1) evaluate cognitive functions of the three groups; (2) compare volumes of the left lateral ventricle, right lateral ventricle, third ventricle, fourth ventricle, and whole ventricles (adding bilateral ventricles, third ventricle, and fourth ventricle) among the three groups; (3) evaluate left lateral ventricle-to-brain ratio (LLVBR), right lateral ventricle-to-brain ratio (RLVBR), third ventricle-to-brain ratio (TVBR), fourth ventricle-to-brain ratio (FVBR), and whole ventricle-to-brain ratio (WVBR) among the three groups; and (4) conduct a correlation analysis between ventricular volumes with cognitive and clinical variables.

## Materials and Methods

### Participants

In the current study, 60 subjects were enrolled, including 20 manic PBD patients, 21euthymic PBD patients, and 19 age- and gender-matched HCs. All of the PBD patients were recruited from the clinical psychiatric department in the Second Xiangya Hospital of Central South University, while HCs were enlisted via advertisement. This study was conducted from January 2012 to July 2014.

The inclusion criteria of PBD patients were: (a) having met the Diagnostic and Statistical Manual for Mental Disorders, Fourth Edition (DSM-IV) criteria for BD with current manic and euthymic episodes; (b) manic PBD conformed to hypomania (≥4 days) or mania (≥7 days) and euthymic PBD patients were needed to have experienced a remission period for more than four consecutive weeks prior to the study; (c) 10–18 years old; and (d) right-handedness.

Exclusion criteria for all of the subjects were: (a) score of intelligence quotient (IQ) <80; (b) contraindications for MRI scanning, including the presence of a pacemaker, artificial metal heart valves, aneurysm clip, other foreign metal matter in the body, or claustrophobia; (c) history of alcohol or substance abuse in the 2 months prior to the study; (d) history of cranial trauma; and (e) other psychiatric illnesses, including schizophrenia, attention-deficit hyperactivity disorder, or anxiety disorder.

The study obtained approval from the Ethics Committee of the Second Xiangya Hospital of Central South University. All of the participants and at least one parent or legal guardian signed informed consent documents.

### Clinical and Cognitive Evaluation

The children and at least one parent or legal guardian were assessed employing Affective Disorders and Schizophrenia for School-Age Children-Present and Lifetime Version (K-SADS-PL) (23) by clinical interviews. The diagnosis of PBD was carried out by two senior child psychiatrists who had extensive clinical experience of diagnosis in PBD. Demographic, clinical, and neurocognitive information was collected for all subjects. Clinical scales, including IQ, Young Mania Rating Scale (YMRS), Mood and Feelings Questionnaire (MFQ) scores, onset age, illness duration, and onset frequency, were recorded. YMRS (24) and MFQ (25) were used to evaluate the severity of manic and depressive symptoms, respectively.

Cognitive functions were measured by Stroop color-word test (SCWT), trail making test (TMT), and digit span subtest (DST). SCWT was utilized to examine processing speed, perceptual conversion, selective attention, and inhibition of habitual response patterns and sensibility for plasticity of mental control and response (26, 27). SCWT fell into three categories: word reading test (SCWT-A), color naming test (SCWT-B), and color word interference (SCWT-C). In SCWT-A, the subjects were asked to name the word printed on a white piece of paper (e.g., the word “red” printed in black) as quickly as possible. In SCWT-B, words printed in three colors were presented (e.g., “red” printed in red), and the subjects were asked to name the color as quickly as possible. In SCWT-C, the subjects were asked to name the color of words whose meanings were different from the ink color (e.g., “blue” printed in red) as quickly as possible, while trying to ignore the meaning of the word. Participants were requested to finish every test within 45 s, and the number of correct answers was recorded as the SCWT score. TMT was used to assess the participants' abilities in mental processing speed, attention, cognitive order, spatial perception, eye and hand coordination, and flexibility in thinking. TMT was divided into TMT-A and TMT-B. In TMT-A, 25 consecutive numbers surrounded by circles were randomly shown on a paper, and subjects were asked to sequentially connect each circle in ascending order (e.g., 1-2-3…) as quickly as possible. In TMT-B, 25 consecutive numbers and 25 letters enclosed by circles were randomly displayed on a piece of paper, and participants were asked to alternately link every circle by numerical ascending order and alphabetical order (e.g., 1-a-2-b-3-c…). The score of TMT was the time taken to complete the task. DST is a part of the Wechsler scale of intelligence and it is utilized to test short-term memory attention, concentration, and memory. It includes forward DST (DST-F) and backward DST (DST-B). The subjects were required to repeat a series of random numbers from 1–9 in sequential order (DST-F) and in reverse order (DST-B) after hearing them. The DST score was recorded as the maximum number of digits that the subject was able to repeat correctly.

### MRI Acquisition and Processing

#### MRI Scan

For the study, MRI scans were conducted using a Siemens 3.0T Trio scanner (Siemens, Munich, Germany). During the MRI scanning, foam pads were placed on two sides of the heads of the subjects to restrict head motion, and cotton earplugs were used to reduce noise and protect the hearing of the subjects. Every subject was requested to stay awake with eyes closed and not to think specific thoughts during the MRI scan. T1-weighted images were obtained by employing three-dimensional magnetization-prepared rapid acquisition gradient echo (3D MPRAGE) protocol. Acquisition parameters covering the whole brain included: repetition time (TR) = 2,300 ms, echo time (TE) = 2.98 ms, inversion time = 900 ms, thickness = 1 mm, gap = 0 mm, field of view (FOV) = 256 mm × 256 mm, matrix = 256 × 256, and flip angle = 9°.

#### Image Processing and Calculation of Volumes

T1-weighted images were processed by FreeSurfer 6.0 software (http://surfer.nmr.mgh.harvard.edu/). Ventricular volumes and total intracranial volume (TIV) were obtained by recon stream (“recon-all”). The procedure included steps such as: (a) Motion correction was performed to reduce the effect of head movement during scanning; (b) Skull stripping was conducted to remove the skull and extract the brain; (c) Talairach transformation was carried out for affine transformation from the original volume to the MNI305 atlas; (d) Intensity normalization was conducted to reduce the intensity difference in the same tissue due to a non-uniform magnetic field or other factors; (e) The brain was divided into gray matter, white matter, and cerebrospinal fluid (CSF); and (f) A transform was created in linear transform array format. In addition, the tessellation of the boundary was conducted between white and gray matter. To ensure proper segmentation of ventricles, a trained physician re-inspected all segmented ventricle borders and corrected them manually, if required. The volumes of five ventricles, namely, the bilateral ventricles, third ventricle, fourth ventricle, and whole ventricles, were used for analysis in the current study. Ventricle-to-brain ratio (VBR) was the value of dividing ventricle volume by TIV, and then multiplying by 100 (22). LLVBR, RLVBR, TVBR, FVBR, and WVBR were calculated for each subject.

### Statistical Analysis

Statistical analysis was conducted using IBM SPSS (version 25.0, Armonk, NY, United States). The Pearson chi-square test was employed to evaluate categorical variables, including gender, psychotic symptoms, BD subtype, and familial BD history. Parametric tests were used when data satisfied both normal distribution and homogeneity of variance at the same time. When data did not satisfy normal distribution or homogeneity of variance, non-parametric tests were used. Shapiro-Wilk test and Levene's test were used to assess the normality of distribution and homogeneity of variance.

Age and gender were deemed as covariates in the comparison of cognitive variables and VBR. TIV, age, and gender were considered as covariates in the comparison of ventricular volumes. One-way ANCOVA or Kruskal-Wallis H test was applied for comparison of cognitive variables, ventricular volumes, and VBR among the three groups. False discovery rate (FDR) correction (http://www.sdmproject.com/utilities/?show=FDR) was used in the comparison of the main effects. Two-sample *t*-test or Kruskal–Wallis H test was employed for *post hoc* analysis and Bonferroni correction was used for correction of multiple comparisons. Because the sample size in each group was relatively small, statistical power (Cohen's *d*) was provided to show differences between the PBD patients and the HCs. Pearson correlation analysis was employed to assess correlations between ventricular volumes and cognitive and clinical indices, with age, gender, and TIV as covariates. Significant level for all of the analyses was set as *p* < 0.05. Normal distributed data were expressed as mean ± standard, and non-normal distributed data were reported as median and inter-quartile range [M(QU-QL)].

## Results

### Demographic and Clinical Information

As shown in Table 1, there was no significant difference among the three groups in gender, age, education, IQ, or MFQ scores. YMRS scores showed a significant difference among the three groups. No significant difference was found between manic and euthymic patients in onset age, illness duration, onset frequency, psychotic symptoms, BD subtype, and familial BD history.

**Table 1:** Demographic and clinical information of the participants.

**Characteristics**	**Manic-PBD**	**Euthymic-PBD**	**Healthy controls (*n* = 19)**	***H/U/T/χ^2^***	***P***
	**(*n* = 20)**	**(*n* = 21)**			
Gender (male/female)	7/13	11/10	7/12	1.54^#^	0.463
Age (years)^b^	14.50 (14.00–16.00)	15.00 (14.00–17.00)	15.00 (12.00–15.00)	3.82^&^	0.148
Education (years)^b^	8.00 (7.00–9.00)	8.00 (7.00–10.05)	8.00 (6.00–9.00)	1.97^&^	0.374
IQ^b^	97.00 (90.50–114.75)	111.00 (99.00–114.50)	106.00 (100.00–113.00)	3.58^&^	0.167
YMRS scores^b^	34.50 (29.25–40.00)	6.00 (4.00–7.00)	3.00 (2.00–5.00)	43.17^&^	*** <0.001***
MFQ scores^b^	7.00 (5.25–8.75)	7.00 (2.50–10.00)	6.00 (3.00–9.00)	0.84^&^	0.657
Onset age (years)^a^	14.15 ± 1.87	13.57 ± 2.06	-	0.94^∧^	0.354
Illness duration (months)^b^	12.00 (6.00–17.00)	13.00 (11.50–39.00)	–	139.50^~^	0.065
Onset frequency (times)^b^	3.00 (2.00–3.75)	3.00 (2.00–5.50)	–	190.50^~^	0.790
Psychotic symptoms (yes/no)	9/11	12/9	–	0.61^#^	0.437
BD-I/BD-II	16/4	13/8	–	1.62^#^	0.203
Familial BD history (yes/no)	7/13	6/15	–	0.20^#^	0.658
Medications					
Lithium	9 (45%)	8 (38%)	–	–	–
Valproate	10 (50%)	14 (67%)	–	–	–
Atypical antipsychotics	14 (70%)	16 (76%)	–	–	–
Antidepressants	3 (15%)	–	–	–	–

a *mean ± standard deviation*;

b *Median (range)*.

# *Pearson chi-square test*;

& *Kruskal-Wallis H test*;

∧ *Two-sample t-test*;

~ *Mann–Whitney U test*.

*IQ, intelligence quotient; YMRS, Young Manic Rating Scale; MFQ, Mood and Feelings Questionnaire; BD-I, bipolar disorder type I; BD-II, bipolar disorder type II*.

*Bold values indicated significant difference with p < 0.05*.

### Cognitive Variables Analysis

As shown in Table 2, significant differences of cognitive variables among the three groups were found in SCWT-A, SCWT-B, SCWT-C, and DST-B. In comparison with the HCs group, the manic group and the euthymic group presented reductions of score in SCWT-A, SCWT-B, SCWT-C, and DST-B.

**Table 2:** Cognitive variables in Manic-PBD, Euthymic-PBD, and Healthy controls.

						***post hoc***								
**Cognitive scales**	**MPBD**	**EPBD**	**HCs**	**Main effect**		**MPBD** **<** **HCs**			**EPBD** **<** **HCs**			**MPBD** **<** **EPBD**		
				***F/H***	***P-value***	***T/H***	***P*-value**	***d***	***T/H***	***P*-value**	***d***	***T/H***	***P*-value**	***d***
SCWT-A^e^	54.63 ± 15.32	53.38 ± 13.92	66.00 ± 12.26	11.25^b^	***<0.001***	3.72^c^	***0.001***	1.23	4.44^c^	***<0.001***	1.47	−0.63^c^	1.000 (MPBD > EPBD)	−0.19
SCWT-B^f^	74.00 (56.00–88.00)	76.00 (57.00–80.50)	89.00 (81.00–94.00)	21.04^a^	***<0.001***	20.95^a^	***0.001***	1.48	22.75^a^	***<0.001***	1.70	1.80^a^	1.000	0.06
SCWT-C^e^	29.74 ± 7.05	31.57 ± 8.98	40.74 ± 9.43	14.75^b^	***<0.001***	4.93^c^	***<0.001***	1.62	4.48^c^	***<0.001***	1.32	0.58^c^	1.000	0.20
TMT-A^e^	39.42 ± 11.90	38.76 ±12.79	29.74 ± 9.63	2.97^b^	0.083	-	-	-	-	-	-	-	-	-
TMT-B^f^	81.00 (70.00–98.00)	90.00 (67.50–119.00)	69.00 (63.00–93.00)	1.32^a^	0.518	-	-	-	-	-	-	-	-	-
DST-F^f^	8.00 (7.00–9.00)	8.00 (8.00–9.00)	9.00 (8.00–10.00)	3.88^a^	0.168	-	-	-	-	-	-	-	-	-
DST-B^e^	4.42 ± 1.17	4.67 ± 1.62	5.95 ± 1.68	7.79^b^	***0.002***	3.55^c^	***0.002***	1.21	3.31^c^	***0.005***	0.95	−0.33^c^	1.000	0.11

a *Kruskal-Wallis H test*;

b *One-Way ANCOVA*;

c *Two-sample t-test*;

d *adjusted Cohen's d*.

e *mean ± standard deviation*;

f *Median (range)*.

*MPBD, manic pediatric bipolar disorder; EPBD, euthymic pediatric bipolar disorder; HCs, healthy controls; SCWT, Stroop color-word test; TMT, trail making test; DST, digit span test*.

*FDR correction for main effect comparison; Bonferroni correction for post hoc analysis; Presented adjusted p < 0.05 was considered to indicate a significant difference*.

*Bold values indicated significant difference with p < 0.05*.

### Ventricle Volume Analysis

As shown in Table 3, significant differences of ventricular volumes among the three groups were found in five ventricles, namely, the left lateral ventricle, right lateral ventricle, third ventricle, fourth ventricle, and whole ventricles. In comparison with the HCs group, the manic group presented enlarged volumes in the left lateral ventricle, right lateral ventricle, third ventricle, and whole ventricles, and the euthymic group displayed increased volumes in the third ventricle, fourth ventricle, and whole ventricles. No difference in ventricle volume was found between the manic and euthymic PBD patients.

**Table 3:** Ventricular volumes in Manic-PBD, Euthymic-PBD, and Healthy controls.

						***post hoc***								
**Regions**	**MPBD**	**EPBD**	**HCs**	**Main effect**		**MPBD** **>** **HCs**			**EPBD** **>** **HCs**			**MPBD** **<** **EPBD**		
				***F/H***	***P-value***	***T/H***	***P*-value**	***d***	***T/H***	***P*-value**	***d***	***T/H***	***P*-value**	***d***
Left lateral ventricle^f^	6.52 (5.37–9.97)	6.85 (5.75–9.91)	5.57 (4.17–6.60)	7.90^a^	***0.024***	14.09^a^	***0.035***	0.93	13.12^a^	0.053	0.88	0.97^a^	1.00	0.22
Right lateral ventricle^f^	5.61 (4.87–7.83)	5.73 (4.36–7.64)	4.06 (3.32–6.11)	8.60^a^	***0.023***	15.26^a^	***0.019***	0.91	12.92^a^	0.059	0.84	2.34^a^	1.00	0.22
Third ventricle^e^	1.22 ± 0.43	1.28 ± 0.30	0.94 ± 0.21	5.19^b^	***0.020***	2.79 ^c^	***0.022***	0.90	2.83 ^c^	***0.019***	1.08	0.01^c^	1.00	0.00
Fourth ventricle^f^	2.04 (1.60–2.34)	2.10 (1.77–2.72)	1.56 (1.39–1.83)	7.39^a^	***0.025***	12.52 ^a^	0.076	0.81	13.71 ^a^	***0.039***	0.99	1.19 ^a^	1.00	0.12
Whole ventricles^f^	15.58 (13.15–21.63)	16.50 (12.94–21.83)	11.12 (10.11–15.57)	10.83^a^	***0.020***	16.57^a^	***0.009***	1.02	15.27^a^	***0.017***	1.08	1.30^a^	1.00	0.20

a *Kruskal–Wallis H test*;

b *One-Way ANCOVA*;

c *Two-sample t-test*;

d *adjusted Cohen's d*.

e *mean ± standard deviation*;

f *Median (range); nit: cm^3^*.

*MPBD, manic pediatric bipolar disorder; EPBD, euthymic pediatric bipolar disorder; HCs, healthy controls*.

*FDR correction for main effect comparison; Bonferroni correction for post hoc analysis; Presented adjusted p < 0.05 was considered to indicate a significant difference*.

*Bold values indicated significant difference with p < 0.05*.

### Ventricle-To-Brain Ratio Analysis

As shown in Table 4, no significant difference of VBR among the three groups was found in five ratios: LLVBR, RLVBR, TVBR, FVBR, and WVBR.

**Table 4:** Ventricle-to-brain ratio in Manic-PBD, Euthymic-PBD, and Healthy controls.

				**Main effect**	
**Regions**	**MPBD**	**EPBD**	**HCs**	***F/H***	***P-value***
LLVBR^d^	0.50 (0.36–0.66)	0.48 (0.39–0.66)	0.35 (0.28–0.47)	5.17^a^	0.076
RLVBR^c^	0.45 ± 0.17	0.42 ± 0.13	0.31 ± 0.10	4.04^b^	0.057
TVBR^d^	0.08 (0.06–0.10)	0.09 (0.08–0.10)	0.07 (0.05–0.08)	6.78^a^	0.057
FVBR^d^	0.14 (0.11–0.16)	0.14 (0.12–0.18)	0.11 (0.10–0.13)	5.30^a^	0.076
WVBR^d^	1.10 (0.90–1.47)	1.12 (0.93–1.45)	0.82 (0.68–1.05)	8.21^a^	0.057

a *Kruskal–Wallis H test*;

b *One-Way ANCOVA*.

c *mean ±standard deviation*;

d *Median (range)*.

*MPBD, manic pediatric bipolar disorder; EPBD, euthymic pediatric bipolar disorder; HCs, healthy controls; LLVBR, left lateral ventricle-to-brain ratio; RLVBR, right lateral ventricle-to-brain ratio; TVBR, third ventricle-to-brain ratio; FVBR, fourth ventricle-to-brain ratio; WVBR, whole ventricle-to-brain ratio*.

*FDR correction for main effect comparison; Presented adjusted p < 0.05 was considered to indicate a significant difference*.

### Correlation Analysis

Pearson correlation analysis was performed between ventricular volumes with clinical and cognitive indices. No significant correlation (*p* > 0.05) was found between ventricular volumes or VBR with clinical and cognitive indices in either the manic PBD groups or euthymic PBD groups.

## Discussion

In the current study, cognitive function and brain ventricle volumes were evaluated among manic PBD patients, euthymic PBD patients, and healthy adolescents. When compared with HCs, the manic and euthymic PBD patients showed decreased scores of SCWT and DST-B. In terms of ventricle volume comparison, larger bilateral ventricle volumes in the manic PBD group, larger fourth ventricle volume in the euthymic PBD group, and enlarged third ventricle and whole ventricle volumes were found in both PBD groups.

In the current study, the manic as well as euthymic PBD patients exhibited significant impairment in cognitive tests compared with the HCs. The damage in cognition included poor performance in tests of processing speed, concentration and attention, working memory, and executive function. Specifically, the manic and euthymic PBD patients performed worse on SCWT and DST-B compared with the HCs. SCWT is a test of processing speed, cognitive flexibility, and response inhibition. Deficits of SCWT may imply that the manic and euthymic PBD subjects were damaged in brain areas such as the anterior cingulate cortex, dorsolateral prefrontal cortex, lingual gyrus, and extrastriate cortex, which are involved in executive control, speed of color processing, and word-form processing (28). Current results were in line with those of a previous study, that showed poorer performance of SCWT was discovered in PBD patients than in HCs (29). DST is a test of attention and concentration. A prior study found that DST-B of BD was obviously different than HCs, while no difference was displayed in DST-F between adult BD and HCs (30). Impairment of executive function was found in the manic, depressive, and euthymic BD I patients, including initial reaction, inhibition control, and strategic thinking (31). It had been reported that euthymic PBD patients exhibited impairment of cognitive functions, including verbal learning and working memory (32). That more severe damage of the DST-B in PBD patients than that of the HCs shown in the current study suggests a related dysfunction of executive control of phonological information in the manic and euthymic PBD patients. It is worth noting that manic and euthymic PBD patients exhibited similar cognitive damage, and no significant difference in SCWT and DST was found between the two groups. This may imply that although euthymic PBD patients expressed relatively stable mood states, they had cognitive impairment similar to that of manic patients, indicating impairment of brain structures and function in related cognitive circuits in euthymic PBD patients.

Bilateral ventricles' volumes were found to be increased in manic PBD compared to those of HCs in the study. No significant difference of lateral ventricles' volumes was found between euthymic patients and HCs, or between euthymic and manic patients. Similar results were reported in a study of adult BD, which showed the larger lateral ventricular volumes were related to the number of manic episodes in adult BD (10). It had been shown that patients with 22q11.2 deletion syndrome (22q11DS) exhibited enlarged lateral ventricle volumes compared with HCs (33). The 22q11DS, which affected multiple genes involved in neurodevelopment, was also reported to be related with early-onset BD (34). This may imply that the 22q11DS is a genetic risk factor for BD. In animal experiments with mice, ongoing dysregulation of neural cell adhesion molecules in neuropsychiatric disorders led to an increase in lateral ventricular volumes, and affected cognitive function via learning, synaptic plasticity, and long-term potentiation influences (35).

Concerning the third ventricle, we found that third ventricular volume in manic and euthymic PBD patients was larger than that of the HCs, and that third ventricle volume was not an obvious discrepancy between manic and euthymic patients. The result in PBD was not in line with that of previous studies in which adult BD patients were grouped into those with and without psychotic forms, and enlarged third ventricle volume was only discovered in psychotic BD patients (36). Increased third volume may be related to the surrounding anatomical boundary, such as decreased volumes of the thalamus and hypothalamus (5). In post-mortem BD research, total selective neurons robustly decreased by about 50% in the paraventricular nucleus of hypothalamus (37). In other ongoing research, we found a decrease of thalamic volume in the same PBD samples, matching the increased third ventricular volumes in the current study.

We found that the fourth ventricle of euthymic PBD was markedly greater than that of HCs. The shape and size of the fourth ventricle was believed to be influenced by the surrounding brainstem structure. The variation of CACNA1C gene was reported to increase the risk of mental diseases, particularly BD, and to affect brainstem volume (38). Few previous studies focused on the volume of the fourth ventricle in PBD. However, two studies reported larger volumes of the fourth ventricle in schizophrenia (39, 40). To some extent, previous findings elucidated that BD and schizophrenia partially shared heritability and showed some similar clinical features (1, 41, 42). In mental disorders, the region of the brainstem reticular activating system may play an essential role in supporting attention, but it was easily influenced by environmental and genetic influences, and these influences may be the etiology of attention impairment (43, 44). These research studies disclosed that impaired subcortical structures near the fourth ventricle likely indicated pathophysiology of sustained inattention in psychiatric disorders. Considering that euthymic PBD patients also show daily clinical presentation of inattention, the increased fourth ventricular volume exhibited in the current study may indirectly indicate a similar inattention mechanism in euthymic PBD with that of schizophrenia.

In the current study, increased whole ventricular volumes were found in manic and euthymic PBD patients compared to those of HCs. The differences in RLVBR, TVBR, and WVBR among the three groups closely reached statistical significance and the three ratios in manic and euthymic PBD patients were larger than those of the HCs, as shown in Table 4. Previous studies found increased LVBR in older BD patients (45) or else no alterations of LVBR and TVBR in adult BD patients (46). Nevertheless, two studies did not find the volumetric alteration of ventricles in adult BD (47, 48). The underlying mechanisms for BD seemed different in adults and children (49). Our results regarding whole ventricular volume and VBR provided more clues in the field of PBD.

Several limitations should be acknowledged in the current study. First, the sample size of our study was relatively small, which may cause an inability to observe some subtle changes in the ventricular volume or significant correlations between alterations of ventricular volume and cognitive and clinical indices. In our future work, much more data will be extracted to make the study more convincing. Second, medical treatment was a potential obstruction in interpreting the present results. Some studies, however, reported no significant effect of medication in behavioral (50) and neural differences between patients and HCs (51). Third, the ventricular volume corrected by the two methods in the present study, that is, the regression-based residuals in calculating brain volume (the residual method, Table 3) and the region-to-ICV ratio (the proportion method, Table 4), were different. After FDR correction, the significant differences in VBR among the three groups disappeared. This may be due to the influence of random errors, which were combined in the numerator and denominator, and then the sources of the error were hidden in the final ratio values (52). In future work, some improvement may be needed to reduce the influence of the random errors.

In the current study, enlarged ventricle volumes were found in manic and euthymic PBD patients compared with those of HCs, which may provide some new clues to brain structural impairment of child and adolescent BD patients. No significant difference of cognitive performance and ventricle volume was observed between manic and euthymic PBD patients, which may imply that the alterations are not specific to mood state. However, when in remission, euthymic PBD patients behaved with similar abnormalities in cognition and ventricle volume as the manic patients, which may indicate damage of the brain structure and function of corresponding brain circuits in euthymic PBD patients.

## Data Availability Statement

The raw data supporting the conclusions of this article will be made available by the authors, without undue reservation.

## Ethics Statement

The studies involving human participants were reviewed and approved by the Ethics Committee of the Second Xiangya Hospital of Central South University. Written informed consent to participate in this study was provided by the participants' legal guardian/next of kin.

## Author Contributions

QJ and GL: conception and study design. WG and LS: data collection or acquisition and clinical support. DC and LK: statistical analysis. YG and JQ: interpretation of results. LK and WC: drafting the manuscript or revising it critically for important intellectual content. All authors: approval of the final version to be published and agreement to be accountable for the integrity and accuracy of all aspects of the work. All authors contributed to the article and approved the submitted version.

## Conflict of Interest

The authors declare that the research was conducted in the absence of any commercial or financial relationships that could be construed as a potential conflict of interest.
